# An incarcerated appendix and the ileocecum within a left inguinal hernia in an infant

**DOI:** 10.1186/s40792-015-0064-y

**Published:** 2015-07-31

**Authors:** Fumiya Yoneyama, Hideaki Tanaka, Kentaro Ono, Takato Sasaki, Takahiro Jimbo, Chikashi Gotoh, Toru Uesugi, Yasuhisa Urita, Toko Shinkai, Hajime Takayasu, Natsuki Imoto, Kouji Masumoto

**Affiliations:** Department of Pediatric Surgery, Faculty of Medicine, University of Tsukuba, 1-1-1 Tennnoudai, Tsukuba, Ibaraki 305-8575 Japan; Department of Pediatrics, Ryugasaki Saiseikai Hospital, Ryugasaki, Ibaraki Japan

**Keywords:** Amyand’s hernia, Left sided, Children, Inguinal hernia

## Abstract

An 8-month-old boy with a left-sided incarcerated inguinal hernia involving the appendix, cecum, and terminal ileum was successfully managed via an inguinal approach during an emergency operation. A mobile cecum seemed to have contributed to the left-sided incarceration. Only 13 similar cases with the left-sided Amyand’s hernia have been reported in the literature.

## Background

It has been reported in various series that the incidence of incarceration of an inguinal hernia in children ranges from 12 to 17 % and is highest during the first year of life, decreasing thereafter [1]. The contents of the hernia sac may comprise the small bowel, cecum, appendix, omentum, or ovary and fallopian tube [2], and incarceration occurs more often on the right side (82 %) [3].

We herein report a rare case of an incarcerated left-sided inguinal hernia involving the appendix, cecum, and terminal ileum.

## Case presentation

An 8-month-old boy presenting with vomiting and swelling of the left inguino-scrotal region, which had been irreducible for 1 day, was referred to our department. The child had a history of a left inguinal hernia when he was 4 months old, which had been observed without surgical treatment. His vital signs were within the normal limits at this presentation. A physical examination revealed a tender, irreducible left-sided inguino-scrotal swelling. His abdomen was slightly distended with no tenderness. An ultrasonographic survey indicated that the hernia involved part of the intestines, the wall of which was quite edematous. The laboratory data were within normal limits, except for the presence of anemia (hemoglobin 8.6 g/dl). An abdominal X-ray showed that there was no abnormal gas in the patient’s left groin or scrotum but showed moderate dilation of the small bowel with gas. After unsuccessful manual reduction, we decided to perform an emergency operation for the incarcerated left-sided inguinal hernia.

**Fig. 1:**
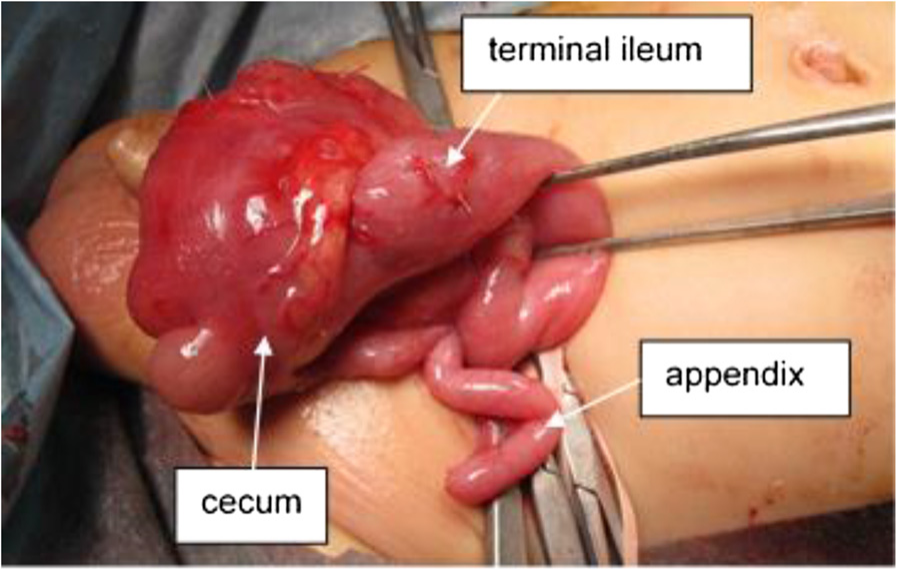
The contents of an incarcerated left inguinal hernia exposed during an emergency operation: the appendix, cecum, and terminal ileum

**Fig. 2:**
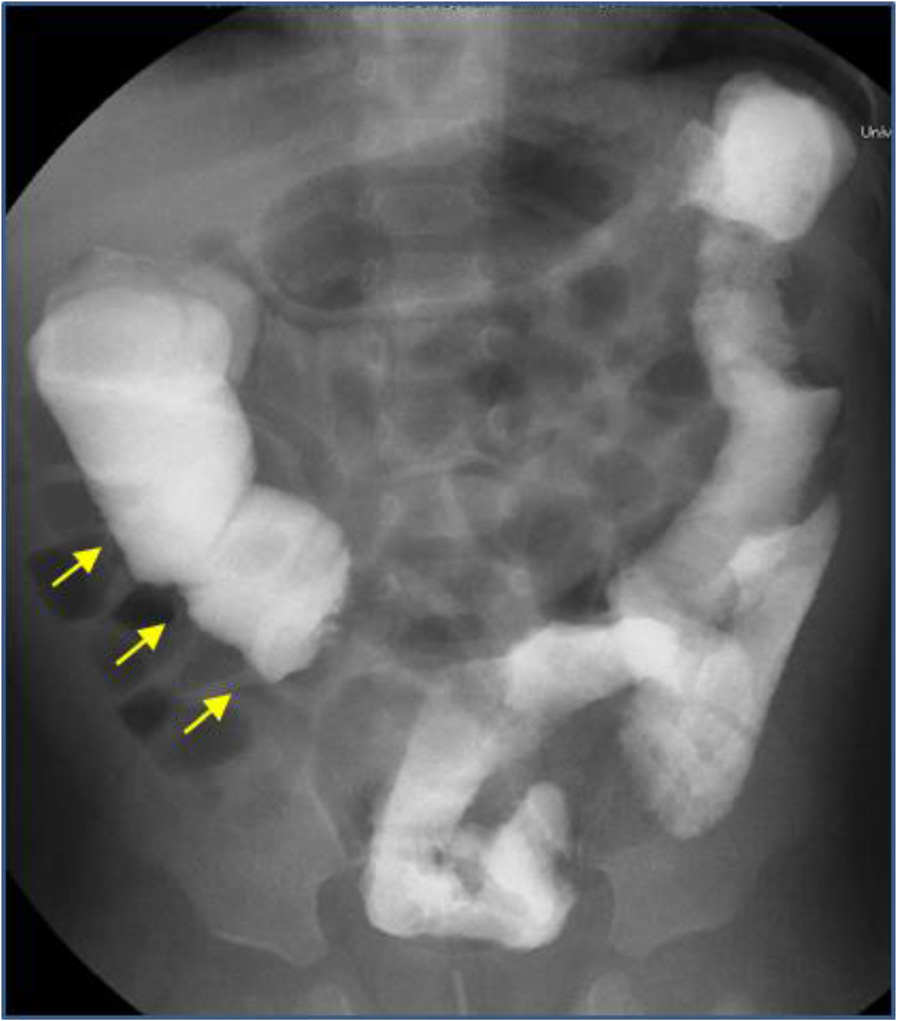
A postoperative contrast study of the colon showed the cecum to be located in the right lower quadrant more centrally than usual (*yellow arrows*), indicating a mobile cecum

After administration of general anesthesia, a transverse skin incision was performed on the left inguinal region. After opening the hernia sac, the appendix, terminal ileum, and cecum were found inside with slight congestion (Fig. 1). The intestines showed no perforation but had several serosal tears, which were repaired using absorbable sutures. Since the appendix was not inflamed, an appendectomy was not performed. After the contents of the hernia were reduced into the abdominal cavity, the hernia sac was dissected free from the spermatic cord, and a left herniorrhaphy was completed with high ligation of the sac (Potts procedure). The patient’s postoperative course was uneventful. His chest-abdominal X-ray did not indicate situs inversus in retrospect. A contrast study of the colon showed that his cecum was more centrally located than usual in the right lower quadrant, which was suggestive of a mobile cecum rather than of intestinal malrotation, judging from the running of the entire colon (Fig. 2). He was discharged in stable condition 2 days after the surgery. A recurrence of an inguinal hernia or testicular atrophy was not identified at his last follow-up visit 1 year postoperatively.

### Discussion

The presence of the appendix in an inguinal hernia sac is referred to as Amyand’s hernia, named after Claudius Amyand, the first surgeon to describe and treat such a case in 1735, simultaneously performing the first appendectomy [4]. Since the hernia contents in our case included the appendix, our case can be categorized as a left-sided Amyand’s hernia. The prevalence of Amyand’s hernia is 0.4–0.6 % among inguinal hernias in the general population and that in children is higher, reaching approximately 1 % [4]. Because of its anatomical position, the appendix is most commonly found in the right hernia sac and can be accompanied by the cecum and/or right colon. Amyand’s hernia can appear on the left side rarely, and only 13 pediatric cases have been described in the literature [5–12].

Table 1 shows the demographics and clinical outcomes of the 14 pediatric cases, including ours, with left-sided Amyand’s hernias. Some authors did not provide detailed information on their cases. To the best of the authors’ knowledge, all of the patients were males whose ages ranged from 2 to 18 months. The presenting symptoms were mainly bilious or non-bilious vomiting, together with tender, irreducible left inguinal swelling. The contents other than the appendix included the cecum in eight cases, the sigmoid colon in one, and the terminal ileum in three. Perforation of the cecum was noted in one case, and serosal tear(s) of the terminal ileum and cecum were noted in each case, all of which were repaired during surgery. Normal appendices in six cases and four cases were preserved and resected, respectively. Inflamed or engorged appendices seen in four cases were all resected. All cases underwent routine herniorrhaphy and had excellent outcomes.

**Table 1:** The demographics and clinical outcomes of pediatric patients with left-sided Amyand’s hernia, including our case

Pt. No	Author	Year	Gender	Age	Presenting symptoms	Contents of hernia	Presence of appendicitis	Underlying condition	Surgical treatments	Outcome
1	Gupta S [5]	2005	Male	9 months	Bilious vomiting, irreducible inguinal swelling	Appendix, cecum	(−)	Mobile cecum	Herniorrhaphy	Uneventful
2	Gupta N [6]	2007	Male	11 months	Bilious vomiting, irreducible inguinal swelling	Appendix, cecum, terminal ileum	(+)	Mobile cecum	Herniorrhaphy, appendectomy	Uneventful
3	Kaymakci A [7]	2009	N/A	N/A	N/A	N/A	(+) in one of three	Mobile cecum	Herniorrhaphy in three, appendectomy in one with appendicitis	Uneventful
4	Kaymakci A [7]	2009	N/A	N/A	N/A	N/A		Mobile cecum		Uneventful
5	Kaymakci A [7]	2009	N/A	N/A	N/A	N/A		Mobile cecum		Uneventful
6	Cankorkmaz L [8]	2010	Male	4 months	Vomiting, irreducible inguinal swelling	Appendix	(−)	Mobile cecum in the one, not known in the other	Herniorrhaphy, appendectomy	Uneventful
7	Cankorkmaz L [8]	2010	Male	2 months	Irreducible inguinal swelling	Appendix	(−)		Herniorrhaphy, appendectomy	Uneventful
8	Khan R [9]	2011	Male	10 months	Scrotum swelling	Appendix, part of the cecum	(+)	Mobile cecum	Herniorrhaphy, appendectomy, another midline incision to explore malrotation, cecopexy	Uneventful except for surgical site infection
9	Singh K [10]	2011	Male	18 months	Bilious vomiting, fever, irreducible inguinal swelling	Appendix, cecum (perforation)	(−)	Mobile cecum	Herniorrhaphy, closure of cecal perforation	Uneventful
10	Singh K [10]	2011	Male	18 months	Scrotum swelling	Appendix, cecum, terminal ileum (serosal tear)	(−)	Not known	Herniorrhaphy, repair of serosal tear	Uneventful
11	Pun A [11]	2013	Male	18 months	Vomiting, irreducible inguinal swelling	Appendix, sigmoid colon	(+)	Mobile cecum	Herniorrhaphy, appendectomy	Uneventful
12	Al-Mayoof [12]	2014	Male	4 months	Vomiting, irreducible inguino-scrotal swelling	Appendix, cecum	(−)	Situs inversus	Herniorrhaphy, appendectomy	Uneventful
13	Al-Mayoof [12]	2014	Male	10 months	Bilious vomiting, fever, irreducible inguinal swelling	Appendix, cecum	(−)	Mobile cecum	Herniorrhaphy, appendectomy	Uneventful
14	Our case	2014	Male	8 months	Bilious vomiting, irreducible inguinal swelling	Appendix, cecum (serosal tears), terminal ileum	(−)	Mobile cecum	Herniorrhaphy, repair of serosal tears	Uneventful

Left-sided Amyand’s hernia is considered to involve associated situs inversus, intestinal malrotation, or a mobile cecum [5]. Among these three possible underlying conditions, 11 left-sided Amyand’s hernias (Table 1) among the 14 cases showed the presence of a mobile cecum, and only 1 case had situs inversus as one of the main causes of the herniation, according to postoperative imaging studies. Regarding the preoperative imaging studies, ultrasonography was used in two cases including ours, but the actual contents could not be detected in either case (data not shown). Contrast-enhanced computed tomography (CT) and a contrast enema have revealed the presence of the ileocecum and/or appendix within left-sided Amyand’s hernias in some adult cases [13–15]. These preoperative imaging studies would be useful for diagnosing Amyand’s hernia and also the presence of situs inversus, intestinal malrotation, or a mobile cecum as possible underlying anomalies, especially in left-sided cases. However, it may not be practical to perform CT or a contrast enema preoperatively to rule out Amyand’s hernia in all pediatric patients with symptoms of an incarcerated hernia, and physical examinations should be enough to prompt emergency operations in those patients.

Surgeons should be aware of and keep in mind the ileocecum as a content of an incarcerated hernia during surgery, even in the left inguinal region, because the ileocecum was included among the contents in almost all cases mentioned in our review. Appendicitis may rarely be encountered in Amyand’s hernia, and the pathophysiology of appendiceal inflammation is considered to involve ischemic events and trauma possibly due to incarceration, adhesion, and/or abnormal location of the appendix [16]. Appendicitis was noted in 4 of the 14 pediatric patients with left-sided Amyand’s hernia (Table 1). The appendicitis patients were treated by appendectomy during an emergency operation. The other ten cases, including our own, had a normal appendix (six appendixes were left and untreated; four were resected). Although an appendectomy needs to be performed in the cases of Amyand’s hernia with appendicitis, according to most reports, this procedure is not believed to be necessary when the appendix, as one of the hernia contents, shows no sign of inflammation. The reasons for hesitation in the resection of the normal appendix are as follows: (1) appendectomy may add to the risk of a surgical site infection [4]; (2) the incision may need to be enlarged to dissect the base of appendix, leading to the weakening of tissues and increasing the probability of a recurrence of inguinal hernia [17]; and (3) it may eliminate the possible use of the appendix in a later operation such as urinary diversion (Mitrofanoff appendicovesicostomy) [7].

One pediatric case with a left-sided Amyand’s hernia underwent an intraoperative abdominal inspection for underlying anomalies via a midline incision [9], which indicated a mobile cecum. Recently, the laparoscopic approach has been demonstrated to offer an advantage over open procedures in patients with an incarcerated inguinal hernia, in that the laparoscopy can visualize the reduction and inspect the incarcerated organ [18]. However, the procedure is not yet commonly performed, and we did not choose the laparoscopic procedure for the treatment of our case because of our limited experience in performing laparoscopic surgery in emergency situations. Successful laparoscopic management of an adult right-sided Amyand’s hernia was reported [19]. Therefore, laparoscopy would be more advantageous for left-sided hernias in children, because it can be used during surgery to confirm if the patient has the abovementioned underlying anomalies. Even if one of those anomalies is detected during a perioperative or intraoperative examination, no treatment is needed for mobile cecum or situs inversus. Intestinal malrotation, however, might require additional surgical intervention such as Ladd’s procedure if the patient has a history of bowel obstruction that may have been caused by volvulus.

## Conclusions

An 8-month-old boy with a left-sided incarcerated inguinal hernia, the contents of which involved a normal appendix, the cecum, and terminal ileum with serosal tears, was successfully managed using an inguinal approach during an emergency operation.

Similar left-sided Amyand’s hernias in children have rarely been reported in the literature. A normal appendix can be preserved in such a situation, but an appendectomy and appropriate repair should be undertaken depending on the presence of infectious and/or traumatic changes among the hernia contents. Underlying anomalies such as a mobile cecum, intestinal malrotation, or situs inversus need to be investigated via imaging studies intra- or postoperatively.

## Consent

Written informed consent was obtained from the patient’s parents for publication of this case report and any accompanying images. A copy of the written consent is available for review by the Editor-in-Chief of this journal.
